# Estimating plant distance in maize using Unmanned Aerial Vehicle (UAV)

**DOI:** 10.1371/journal.pone.0195223

**Published:** 2018-04-20

**Authors:** Jinshui Zhang, Bruno Basso, Richard F. Price, Gregory Putman, Guanyuan Shuai

**Affiliations:** 1 State Key Laboratory of Earth Surface Processes and Resource Ecology, Beijing Normal University, Beijing, China; 2 Department of Earth and Environmental Sciences, Michigan State University, East Lansing, Michigan, United States of America; 3 W.K. Kellogg Biological Station, Michigan State University, East Lansing, Michigan, United States of America; 4 Institute for Future Environments and Science and Engineering Faculty, Queensland University of Technology, Brisbane, Qld, Australia; Wageningen University and Research, NETHERLANDS

## Abstract

Distance between rows and plants are essential parameters that affect the final grain yield in row crops. This paper presents the results of research intended to develop a novel method to quantify the distance between maize plants at field scale using an Unmanned Aerial Vehicle (UAV). Using this method, we can recognize maize plants as objects and calculate the distance between plants. We initially developed our method by training an algorithm in an indoor facility with plastic corn plants. Then, the method was scaled up and tested in a farmer’s field with maize plant spacing that exhibited natural variation. The results of this study demonstrate that it is possible to precisely quantify the distance between maize plants. We found that accuracy of the measurement of the distance between maize plants depended on the height above ground level at which UAV imagery was taken. This study provides an innovative approach to quantify plant-to-plant variability and, thereby final crop yield estimates.

## Introduction

Final maize (*Zea mays*) grain yield results from the conversion of assimilates produced with an adequate supply of nutrients, water, and intercepted light. Plant population, defined as the number of plants per unit of area, is an important variable that substantially affects final grain yield. Adequate distance between plants ensures that sufficient resources are available to maximize yields and limit the intrusion of weeds. The objective of many companies in precision agriculture has been to improve the uniformity of seed planting through development of planters that maximize planting efficiency and the uniformity of plant stands. Early research has shown that row spacing and seeding depth led to high plant-to-plant yield variability and lower yields [1]. More recent research has shown that spatial density of plants is important at both intra-row plant spacing and seedling depth. Recognizing differences between total plant population and plant spatial density are important to understand plant-to-plant yield variability [2–4]. Plant population is a target, set by the producer, to establish an average amount of plants per unit of area in the field. Variability on this large scale is hard to detect unless observations are made from a combine yield monitor. However, plant distance variability on a plant-to-plant basis creates real differences in grain yield across a field. Use of variable seeding rates has been shown to increase production when it is coupled with a maize sustainability rating (CSR) which matches seeding rates to different soil types [5]. Stand reduction after emergence, and therefore increased plant-to-plant variability, has been found to be related to increased yield on a per plant basis until V15 [6]. This is evidenced by an increase in the number of kernels produced per plant. Other factors at planting that can lead to variations in plant-to-plant yield include: planting depth, surface soil crusting, timing in seed germination, and variations in soil physical properties [2].

Unmanned Aerial Vehicles (UAVs) provide a unique tool that is particularly useful in agriculture because of their capacity to capture high resolution imagery at several wavelengths. Multispectral or RGB channel imagery can be converted to vegetation indices such as the Normalized Difference Vegetation Index (NDVI) and excessive green index (EXG) [7]. These indices can effectively differentiate living plants from bare soil, a capacity that is imperative for early season detection of plant spacing [8]. Discrimination of weed seedlings was accurately described through use of a UAV equipped with both visible and multispectral cameras at different altitudes in a sunflower seed field [9]. Several types of multispectral indices are now available, each one with a specific purpose [10–11].

Researchers have focused on directing automated machinery in the field and improving the capacity to detect crop rows in pictures captured by tractor-mounted cameras [12]. Currently there are three main types of crop row identification methods: regression, Hough Transform, and vanishing points. The regression method simulates straight lines from linear regression for crop segmentation [13]. Applicability of this method is limited because it requires prior knowledge of both the number of crop rows and the expected location of each crop row. The Hough Transform is a widely applicable method [14] that projects a set of points from an image’s space to a set of lines in a given parameter’s space, and can convert the line direction in an image’s space to the points detectionA. This method was used to track plant rows in real-time, yielding typical errors at 12.5mm [15]. Despite the robust performance against noise, this method presents two issues of concern: the computation burden from the voting schema, and the definition of a number of detected peaks [16]. To overcome computation redundancy, a Randomized Hough Transform (RHT) algorithm was further tested for crop row detection [17]. This method is not yet able to identify crops under conditions of high weed pressure and crop loss. The vanishing point-based method for crop row detection depends on the elimination of row skeletons [18]. Another method integrates the Hough Transform and vanishing point methods to detect wheat rows [19] by converting a color image to a gray-scale image; segmenting the image to identify plants; and then filtering the entire image to remove weeds or noise and identify crop rows [19]. Additional approaches have been proposed to detect crop rows include stereovision-based or horizontal strips methods [20–22].

Because plant distance is still a critical parameter for crop growth models, it is critical that research focused on how to measure plant to plant distances in a row be completed. UAV systems represent a powerful tool that can be used to collect high-resolution real-time images of cropping systems, and thereby support the calculation of plant interval distances. The objective of this research was to calculate the interval distance between maize plants (hereafter referred to as the maize interval distance) using a UAV to collect imagery with an RGB camera. Herein, we report the results of two different experiments: an initial experiment that measured artificial plants under an indoor pavilion; and a second experiment outdoors that measured maize plants in a farmer’s field.

## Methods

The method we used to calculate plant interval distance with the UAV included three steps: extraction of maize segments with the vegetation indices-based method; calculation of the maize planting row; and extraction of the individual plant position to measure the distance of maize plant interval.

### Basis for plant interval distance using UAVs

UAV systems mounted with visible, multispectral or high-spectral cameras, are efficient in monitoring land surfaces and have dramatically increased the ease of colleing remote sensing data [23–25]. In addition, unlike satellite imagery, UAVs can be used to acquire data on both cloudy and clear days.

Each image captured by a UAV camera represents a single scene which covers a specific extent of the land’s surface. Both the height of the airborne platform (*H*, in meters) and the angle of view (*v*, in radians) of the camera play a decisive role in determining the extent. We define *v* as the diagonal or horizontal direction. The diagonal length of the image captured by the UAV is expressed as:

$$L_{diagonal}=2\tan(\frac{v}{2})H$$ (1)

The prerequisite condition is represented by the bound width (*NS*, the number of columns) and height (*NL*, the number of rows) of the airborne image, in pixels. From Eq (1), the width and height of visual picture, (in meters), is calculated in Eq (2) as:

$$L_{width}=L_{diagonal}\times \cos(\mathrm{atan}(NL/NS))$$

$$L_{height}=L_{diagonal}\times \sin(\mathrm{atan}(NL/NS))$$ (2)

The single maize plant represented by a point in the image at pixel measurement, (x, y) is represented as its coordinates. Thus, the original pixel coordinate (x, y) can be converted into (x_m_, y_m_) at a given meter measurement, where x_m_ and y_m_ are the map position based on the top-left coordinate system. The conversion equation is represented in Eq (3):

$$x_{m}=x\times \frac{L_{width}}{NS}$$

$$y_{m}=y\times \frac{L_{height}}{NL}$$ (3)

This equation serves as the basis to measure the distance between two plants. Point 1 and point 2 coordinates, represented as (x_1_, y_1_) and (x_2_, y_2_), are converted to coordinates formatted as (x_1m_, y_1m_) and (x_2m_, y_2m_) at meter measurement by using Eq (3). The distance (D) between point 1 and point 2 is calculated using Eq (4) as follows:

$$D=\sqrt{{\left(x_{1m}-x_{2m}\right)}^{2}+{\left(y_{1m}-y_{2m}\right)}^{2}}$$ (4)

### Process of plant interval distance calculation

Vegetation indices have been tested for their capacity to effectively identify crops [26]. Among these indices, Excess Green (EXG) index was able to distinguish vegetation from background information (such as soil). The EXG index was introduced in this study to enhance identification of maize and to help extract the individual maize segmentation. EXG is defined in Eq (5) as:

EXG=2g−r−b (5)

$$r=\frac{R^{*}}{R^{*}+G^{*}+B^{*}},g=\frac{G^{*}}{R^{*}+G^{*}+B^{*}},b=\frac{B^{*}}{R^{*}+G^{*}+B^{*}}$$

and R^*^, G^*^ and B^*^ are the normalized RGB values (0,1) calculated from:

$$R^{*}=\frac{R}{R_{\max}},\;G^{*}=\frac{G}{G_{\max}},\;B^{*}=\frac{B}{B_{\max}}$$

where *R*, *G* and *B* are the pixel values of red, green and blue of the original RGB image. *R*_max_ R, *G*_max_ and *B*_max_ are the maximum brightness value for each primary band.

A threshold-based method calculated with the Otsu algorithm provides an efficient way to divide an image into vegetation and background information [26]. That process generates a white-black image that is numerically scaled from 0 to 1. Two main issues were addressed in this study with respect to outlier removal in maize identification. The first issue is “contamination” caused by reflectance of non-maize plants (weeds) growing in the field, or crop residue that may be misidentified as maize. A new operation is applied to further filter the maize parcel collection and remove disturbances from non-maize plants and weeds. By analyzing unique characteristics of maize, such as the plant’s area or shape, we can distinguish it from other types of vegetation. Two thresholds of the area and shape, *T*_area_ and *S*_shape_, were set to identify maize. The methods to set these thresholds are carried out by following criteria in Eq (6):

$$O_{maize}=A_{maize}\cap S_{object}$$ (6)

where A_object_ is the filtered maize parcel collection greater than $T_{area}\times \bar{A_{object}}$; S_object_ is the parcel collection greater than $S_{shape}\times \bar{S_{object}}$, among T_area_ and T_shape_ are the respective thresholds for area and shape of vegetation objects, $\bar{S_{object}}$ and $\bar{A_{object}}$ are average area and shape evaluation calculated from the extracted objects, ∩ is symbol that means *O*_*maize*_ will meet the two previous conditions.

The second issue that should be noted is the process used to merge fragmented segments into individual maize objects. Following the identification process described above, objects with parameters within the filtered threshold are considered maize objects. However, an individual maize plant may be separated due to disturbance from the shade from either maize leaves or stem. The dilation operation is carried out to expand the maize objects to form a buffer region along the original maize object. Maize objects with overlap the buffer boundaries are merged to form an individual maize plant. The structure template setting for the dilation process is determined depending on the prior knowledge based on the approximate distance of maize plant interval which varies according to maize planting scenario.

### Calculation of the centroid of segmentation to locate the position of maize plant

**Fig 1:**
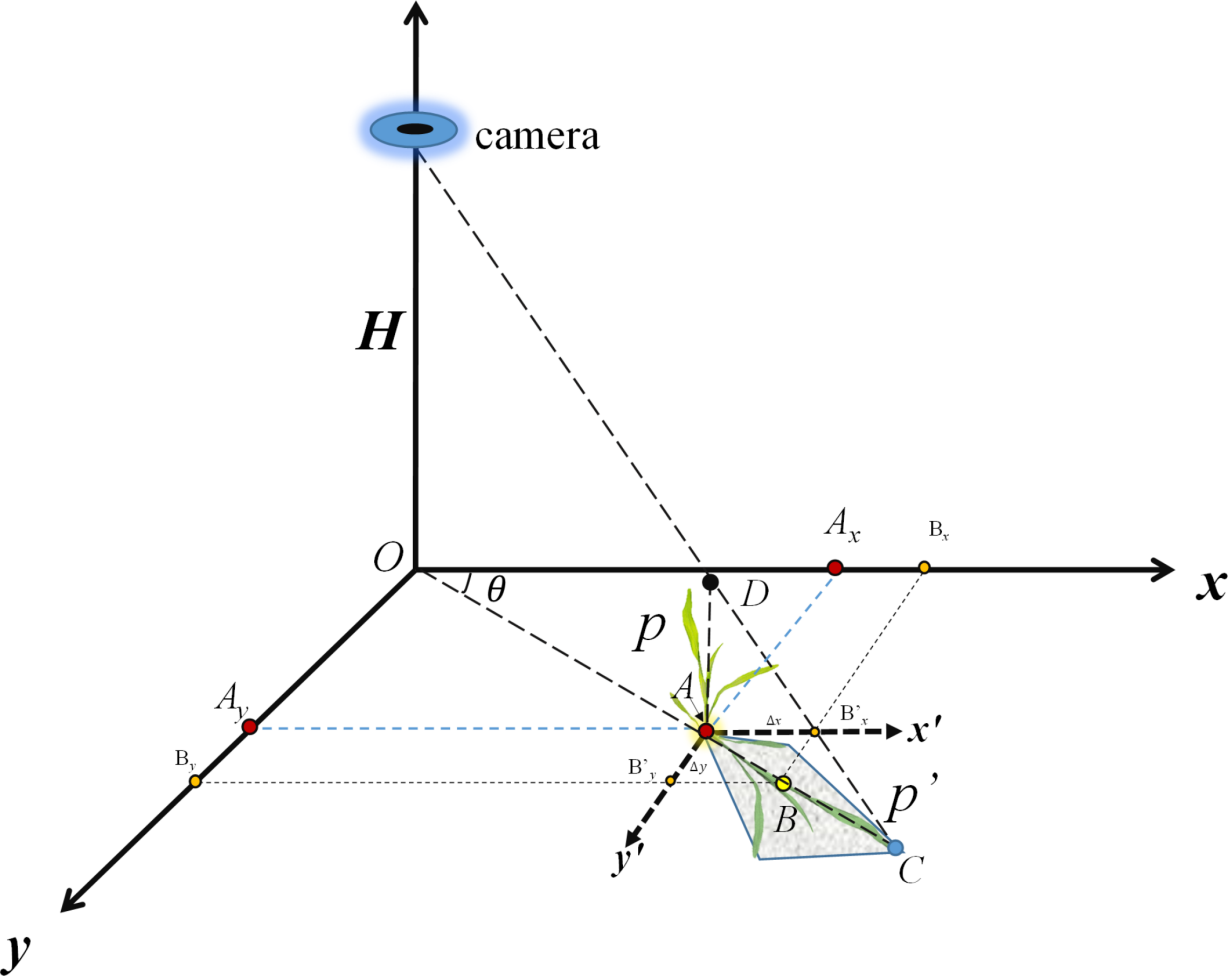
Schematic drawing of the relationship between camera, maize plant and related two-dimensional maize plant projection.

We extracted the centroid point of maize objects from the previous segmentation as a basis for measuring the distance between maize plants. Fig 1 is a schematic of the camera and a plant on the ground which illustrates the relationships between the camera, maize segmentation centroid, and plant height and shows the three-dimensional coordinate system established from this process. The *xOy* plane represents the ground, and the camera above the ground is in the third dimension (z). The original setting for the camera is at nadir, where the camera’s position relative to the ground is directly over the center of the image. The maize plant, *p*, is presumed to be upright; *p*’ is the projected image of *p* observed by the camera. A is the location of the maize plant, p. Our goal is to extract the accurate position of A, the key variable necessary to calculate the interval between maize plants.

The shaded area in Fig 1 is a polygon defined as the maize plant projected to the ground using the camera location as a defining angle. C is the position formed on the ground by the projection of the highest point of the maize plant and the centroid of this projected polygon is point B. The algorithm of the centroid of the maize segment at the pixel scale in *i*_th_ column and *j*_th_ row are formatted in Eq (7) as:

$$centroid_{maize}=\left\{\begin{array}{c} x=\sum_{i=1}^{N}x_{i}/N\\ y=\sum_{i=1}^{N}y_{i}/N \end{array}\right\}$$ (7)

The *centroid*_*maize*_ is the centroid point of the maize segmentation and *x* and *y* are the coordinate positions in *xOy*. The *i* relates to the *i*_th_ pixel in each of the pixel collections within the maize segmentation. The total pixel number of this maize segmentation is *N*.

As seen in Fig 1, A is the actual position at which the maize plant (p) is growing in the field. This position varies widely from point B, the projected centroid, calculated from Eq (3). The difference between these two points is caused by the position of maize *p* compared to the projected perpendicular position of the camera and the camera height (*H*) relative to the ground. If the camera were hung directly over the plant, the centroid of the projected plant (*p’*) would be at a minimum. That is, the distance of AB would be zero without considering the leaves’ randomized distribution. When the camera height is lower relative to the ground, the projected length of *p’* would be larger than if the camera was raised.

Thus, it is essential to know plant height parameter to locate the origination of plants in the field (point A in Fig 1). In this paper, we assumed that the crop grows uniformly within the field under similar biophysical and biochemical conditions. The length of AD (in Fig 1) represents the height of maize. Here, we defined the height of the tallest leaf on the stalk as the height of an individual plant. In addition, the position of point B, the center of the *p*’, can be calculated from Eq (3). Thus, based on the position of B, the known height of the camera (H) and the measured height of the plant (AD), the position of A, the location of individual maize plant, could be obtained.

As shown in Fig 1, Δ*B*_*x*_, Δ*B*_*y*_ are variable coordinates in the *x* and *y* dimensions from the position of point A to point B, calculated from the length of the line formed by these two points (*AB*). The steps below represent the process used to calculate the position of point A.

The relationship between the maize and the camera is described in Eq (8):

$$\frac{AD}{H}=\frac{AC}{OC}$$ (8)

AC and OC can be calculated as:

AC=2AB (9)

OC=OB+BC=OB+AB (10)

Thus, Eq (8) can be transformed as:

$$\frac{AD}{H}=\frac{2AB}{OB+AB}$$ (11)

Then AB can be quantified as:

$$AB=\frac{AD\times OB}{2H-AD}$$ (12)

AD, and H are measured as respective average crop height and camera height, respectively, and considered uniform within one field. OB is the centroid calculated from Eq (3).

The next step is to adjust the position of B to the position of A based on the measurement of AB. From Fig 1, the coordinate of position A (A_x_, A_y_), can be represented based on the B coordinate (B_x_, B_y_), as shown in Eq (13):

$$A_{x}=B_{x}-\left|AB\right|\cos∠x'AC=B_{x}-\left|AB\right|\cos∠\theta$$

$$A_{y}=B_{y}-\left|AB\right|\sin∠x'AC=B_{y}-\left|AB\right|\sin∠\theta$$ (13)

|AB| is the length of the line segment of AB, calculated from Eq (12). ∠*x*'*AC* is the *θ* defined as ∠*BOx*, seen in Fig 1.

#### Detecting planting rows

In our study, a fixed planting width was set for maize planting using large-scale planting equipment. The traditional Hough Transformation-based method for crop rows detection was not adopted because of its enormous computation burden [16]. For one image captured by the camera, the conditions of the direction (*d*) and width (*w*) maize planting ridge were predefined as the prior knowledge to establish the lines for maize rows. From Eq (7), the centroids of individual maize plants were calculated. For one maize row, the center points were approximately adjacent to the ridge.

**Fig 2:**
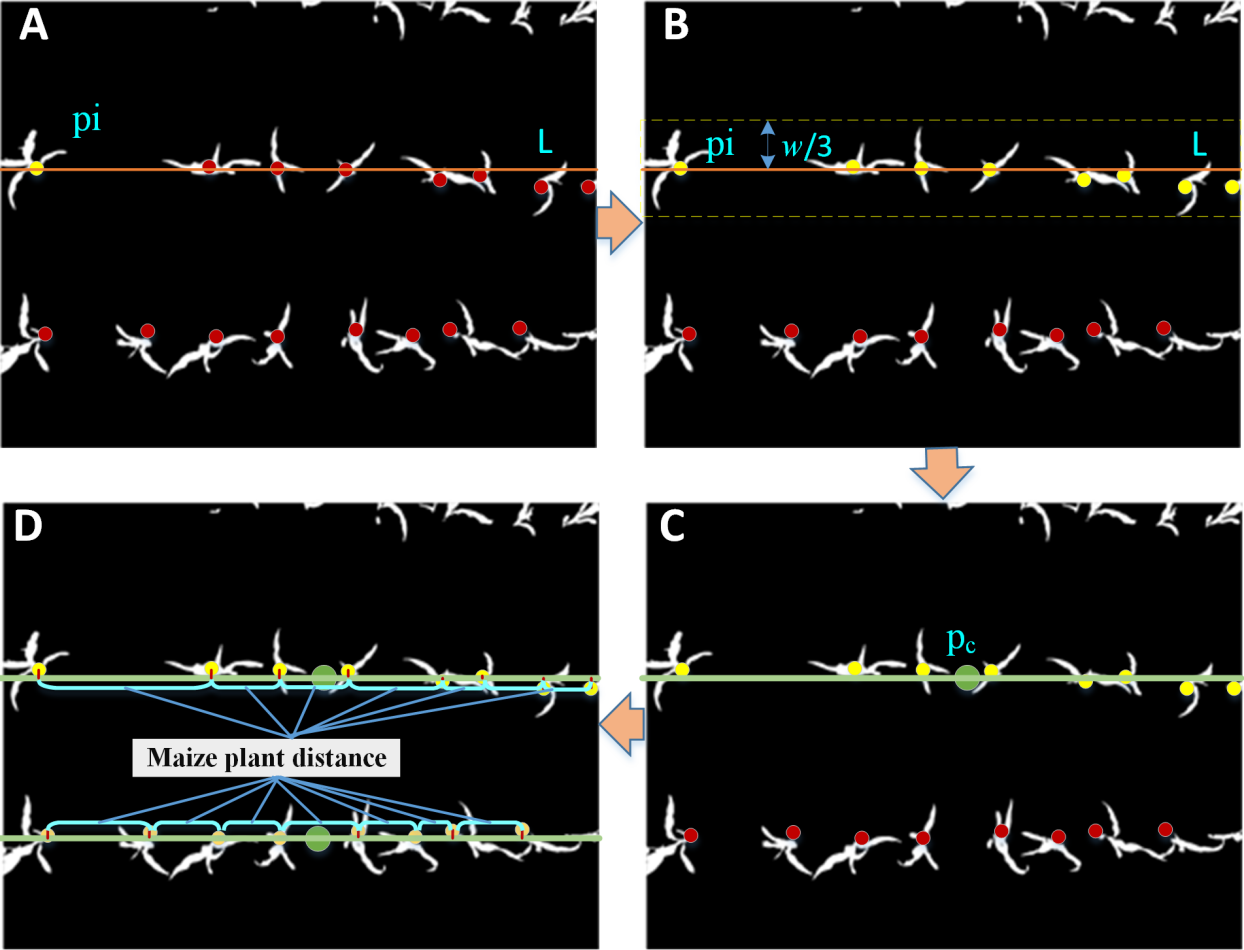
The schema of maize planting rows construction and maize plant distance calculation. (A) Randomly selected adjusted centroids (p_i_). (B) Determination of the buffer design. (C) Calculate the center points (Pc) of the chosen maize point collection. (D) Updated line that crossed P_c_ point with the predefined crop row angle *d*.

Fig 2 illustrates the procedure we used to determine maize planting rows. Initially, one of the adjusted centroids (p_i_) was randomly selected. A line (L) crossing the chosen point was established based on the crop row angle depending on the maize planting practice. Buffer segmentation occurred at certain distances along the established line, L. Determination of the buffer design is a critical design element and should be done carefully. We set the distance as 1/3*w*, which varies from the field planting scenario. Secondly, the buffer region across the area was chosen to intersect maize growing in the same line which allowed the maize point collection to be determined. These are the yellow points (**B**) in Fig 2. Next, the center points (P_c_) of the chosen maize point collection were calculated, in the same manner as Eq (7). Finally, an updated line that crossed Pc point with the predefined crop row angle *d* was made at the maize growing ridge. These three steps were run iteratively to identify the maize growing ridge until all maize points were used.

#### Calculating the distance between maize plants

Based on the derived results of the adjusted points and ridges, the distance between maize plants can be further calculated. The point in the maize object collection is projected into the ridges we have established. All the maize object points are iterative to locate the nearest maize ridge based on the minimum distance criteria. Then, the adjusted point is projected to the corresponding maize ridge (Fig 2D), and the associated perpendicular foot position is determined. Obviously, the maize plant distance is the length between one maize and its neighbor (Fig 2D). In order to calculate this distance correctly, an independent variable of -100 is entered into the inspective function of the maize crop ridge (*y* = *kx* +*b*, where x = -100, this is the linear function as L in Fig 2B). The dependent variable, y, is calculated based on the algebraic relationship. The coordinate pair, (-100, y), is set as the starting point which can be considered as a position reference, and the distance of the collection of foot points perpendicular to the starting point is measured following Eq (4). Thus, for each maize ridge, the distance between the foot point collection and starting points are sorted in ascending order. The pair of object points based on the sequence of distance is determined from the starting position to opposite end of a given segment. Then, the corresponding distance is the distance of maize interval.

### Accuracy assessment

The key focus in this study is to calculate the accurate interval distance between maize plants. The true interval distance was recorded when the artificial maize were assembled in a field-like, but artificial setting. Therefore, an assessment for the maize interval distance estimation can be quantified using the following metric in Eq 14:

$$d_{e}=\left(\frac{\sum_{i=1}^{n}\left|ed_{i}-d_{i}\right|}{n}\right)$$ (14)

where *d*_e_ is the error metric to quantify the degree of the maize interval distance estimation, *ed*_*i*_ is the *i*_th_ estimated interval distance of the maize pairs, *d*_*i*_ is the associated predefined distance of maize pair interval and *n* is the estimated number of plant pairs in the entire image.

*d*_e_is the absolute metric to quantify the estimated distance, which has the limitation to represent the error scale related to the actual maize interval distance. That is, even though *d*_e_ is lower for those rows where the estimated interval between plants is smaller, this doesn’t verify that these results are better than for those rows with larger distances between plants. The metric below was used to analyze error related to the actual maize planting interval distance; *d* is the fixed and predefined distance of the maize planting setting, such as 9cm, 18cm in this experiment:

$$r=d_{e}/d$$ (15)

Obviously, this metric quantifies the error associated with the actual maize planting interval. It would be acceptable if the values of *r* is lower, even when *d*_e_ is large.

Like the traditional accuracy metric bias [27], *d*_t_ (in Eq 16), is used to qualify the accuracy of the estimated result and indicates the under- or over-estimation of the proposed method when compared to ground truth data considering estimated error compensation:

$$d_{t}=\left(\frac{\sum_{i=1}^{n}\left(ed_{i}-d_{i}\right)}{n}\right)$$ (16)

## Experiment 1: Plant distance estimation (artificial maize plant)

**Fig 3:**
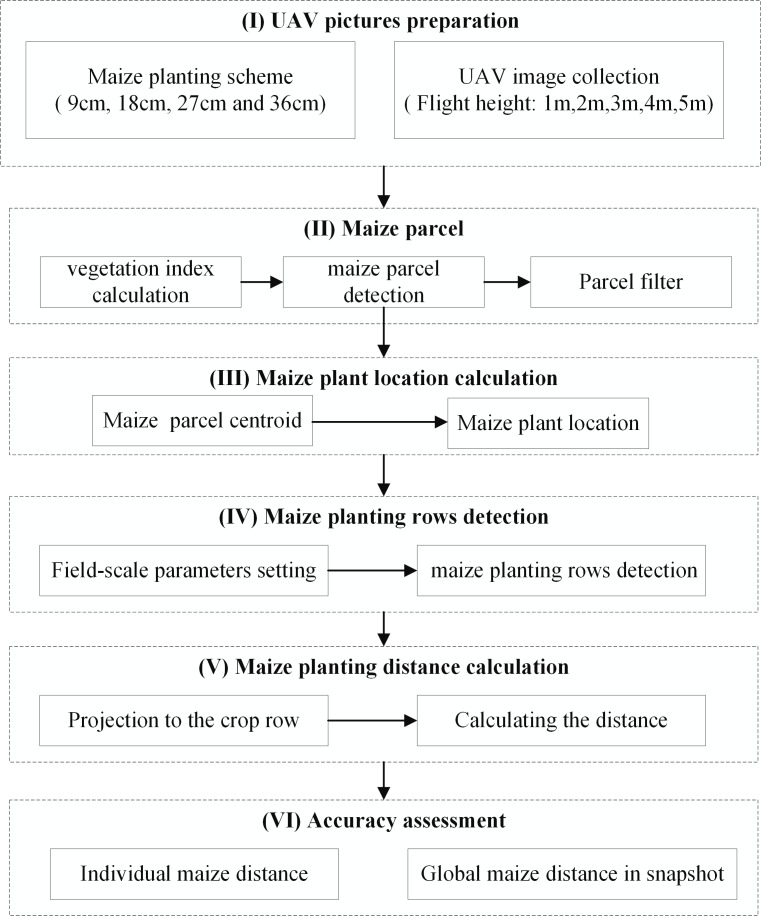
Flowchart of maize planting interval distance calculation.

Fig 3 shows the major steps used to describe the process of calculating maize planting interval distance.

### Maize planting scheme design

In order to avoid the interference of background information, such as crop residue or weeds, we first tested the algorithm indoors under controlled conditions. This simulation was completed April 11, 2016, in the Pavilion for Agriculture and Livestock Education at Michigan State University, a location considered ideal for this experiment. We used artificial plastic plants built to represent young maize plants, on completely bare soil and artificial light that was a combination of halogen bulbs and LED panel fixtures. This combination of light sources was considered adequate to simulate true solar light, and ensures the camera’s ability to collect ample reflectance from the land’s surface for analysis. The artificial maize plants used are made of green plastic, which reflects optical spectra similar to actual maize grown in the natural environment.

It is difficult to analyze images in which maize plants have overlapped leaves, because it is impossible to separate the leaves from one plant from those of another. To avoid this situation, we decided to analyze plants before they reached the V2 growth stage, defined as two fully expanded maize leaves. Plants at this stage are small and distinct from one another and their leaves do not overlap. The interval distance between plants was tested at 9cm, 18cm, 27cm and 36cm to simulate a wide range of planting densities. The most common planting distance used by American farmers is 12 cm. In order to further simulate conditions of farmers’ fields, the leaves of the artificial plants were oriented in random directions and represented proper growth of maize at the V2 stage where leaves emerge in sets of two, 180 degrees from one another. Additionally, the average height of maize at the V2 stage (14cm) was duplicated in our trial. The row spacing of plants in our experiment was 76.5cm, the most common spacing for farmers’ maize fields.

**Fig 4:**
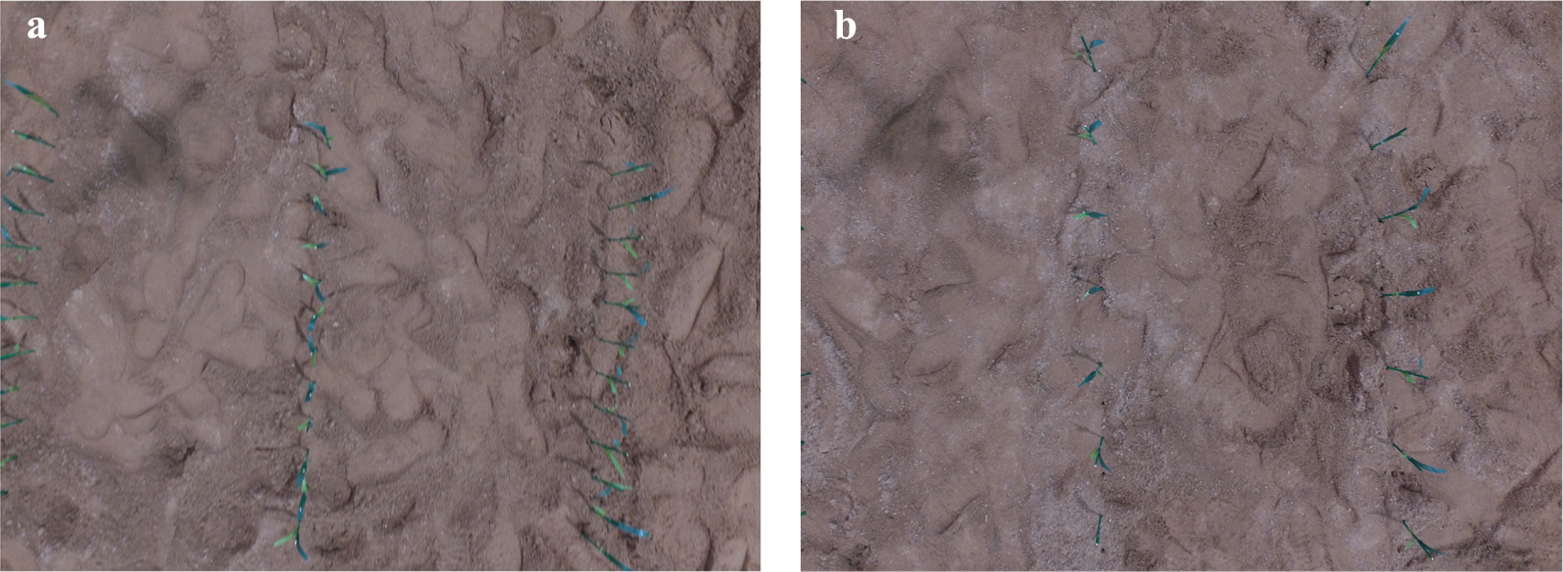
Example of images acquired by UAV platform. (a) S_9cm_1m_. (b) S_18cm_1m_.

Fig 4 shows two examples of the experimental setup which is very similar to what is observed in a famer’s field. In order to concisely describe the different scenarios, we defined a symbol, S_xcm_ym_, where x is the distance of the maize interval and y is the camera’s relative height to ground. For example, S_9cm_1m_ describes a scenario where the distance of the maize interval is set at 9cm and the camera height is 1m.

### UAV image collection

The Phantom 3 Professional, a DJI consumer grade product, was used in this study to acquire high-resolution low altitude images. It is easy to fly with or without GPS assistance, which guarantees uniform flight height, and collects 12-megapixel JPEG files as well as DNG RAW data which enable the user to obtain data efficiently. The view angle of the camera mounted on the flying platform is 72°, a key parameter needed to determine the distance between maize plants as shown in Eq (1).

Considering how the image of maize plants is influenced by the height of the camera, different camera view extents and view angles of 1m, 2m, 3m, 4m, and 5m heights were tested. For each test, the camera was controlled to face the ground vertically (nadir) to ensure that the airborne position was projected into the centroid of the image.

### Maize interval distance extraction

After acquiring the airborne images, an estimate of the distance between maize plants was calculated according to the process previously outlined. The five parameters that were predefined included maize ridge angle, average maize height, buffer distance, two thresholds for maize object area and ratio of perimeter to area.

We defined the maize ridge angle as the angle to the horizontal direction towards the right side in a counterclockwise motion, ranging from zero to 180 degrees. The direction of the maize ridge was oriented vertically in the image to the bounding size as best as possible during image acquisition. As shown in Fig 4A, a small amount of tilt was still present when we took the picture with the DJI. Thus, the maize ridge angle was set for each snapshot separately. We fine-tuned the angle to make the best direction to demonstrate the ridge direction of each individual picture. The average maize height was used to adjust the centroid of the estimated maize object to maize root location. The artificial plant height above the ground was measured, with 10cm as the average value. The thresholds for the maize object area and ratio of perimeter to the area are critical variables to filter the segmented objects into the maize collection (Table 1). Thresholds were set at (0.3) * (average objects’ area) and (0.3) * (ratio of the perimeter to the area).

**Table 1:** Parameters used to detect maize plant location to determine plant distance.

plant height (cm)	buffer distance (cm)	threshold for maize object area	Threshold for ratio of perimete to area
10	4	0.3	0.3

All the objects using the EXG index with 0 thresholds met the criteria for the maize object collection.

## Results

**Fig 5:**
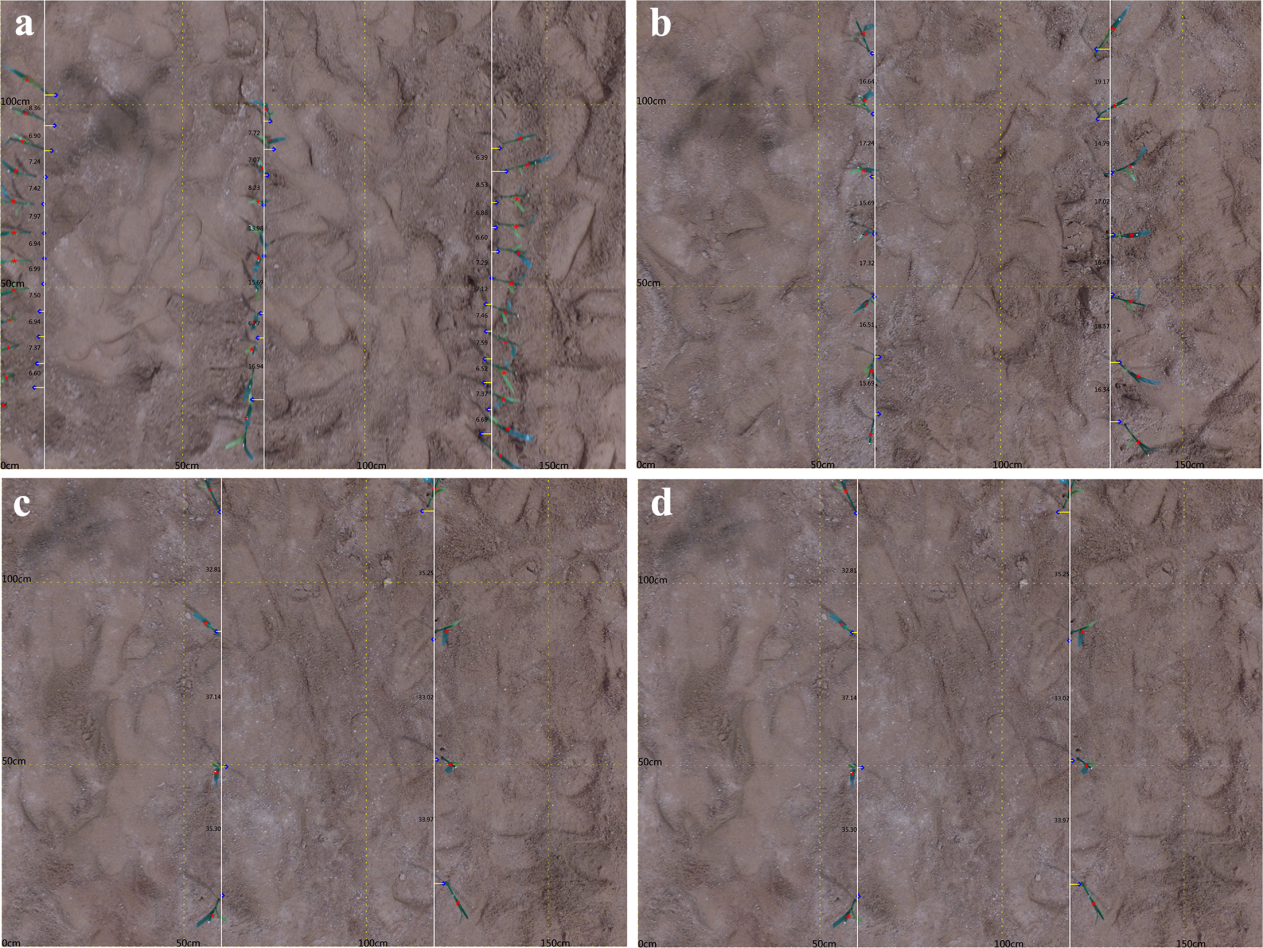
Estimated distance of the maize interval at 1m camera height with different plant distances. (a) 9cm, (b) 18cm, (c)27cm and (d) 36cm.

We calculated the interval distance of maize at several plant densities as shown in the photos below. Red points in Fig 5 were originally calculated as the centroid of individual maize objects. It was clearly observed that all the points were located around the leaf of the maize plant. From the camera position at the centroid of the picture as the relative location, the tilt extent of maize increases with greater distance from the camera, leading to larger deviations of the estimated centroid point (red point) as the location of the maize plant in the ground gets further from nadir. Hence, it was necessary to adjust the centroid of the maize plant to correct for this. The blue points represent the bottom of the maize plants based on the original centroid point. This process completed that task efficiently based on the height of the camera and the plant. The white lines are the estimated maize ridge which was closest to the root of the maize plants. All the estimated points (blue) were then projected into the estimated ridge. The distance of the maize interval was calculated and shown in the space between two maize plants.

For each of the artificial plant scenarios, the quantitative metric, *d*_*e*_, was calculated (see Table 2). These error results are within tolerance levels for crop management. Overall, *d*_*e*_ gradually increased in relation to the height of the camera. The results were acceptable to quantify the maize interval distance throughout the maize interval scales tested (e.g. 18cm to 36cm scale), except for the smallest distance (9cm). The absolute metric, *d*_*e*_, was satisfied with the largest distance of 3.19, but the relative accuracy, *r*, is unfavorable. Relative errors for the 9 cm spacing were greater than 10%, especially when the flight height was increased to 5m where the calculated error was 35.46%. The accuracy for the 9cm spacing was not acceptable for further application because of the large error compared to the actual interval distance of the maize. For *d*_*t*_ to demonstrate the total biases of the whole image, the value was larger or smaller than the actual images values. The *d*_*t*_ showed a similar trend as *d*_*e*_ where large errors occurred in association with larger distance of the maize interval, amongst the value of *d*_*e*_ with parentheses were negative.

**Table 2:** The estimated distance of maize interval (cm).

	9cm			18cm			27cm			36cm		
	*d*_*e*_/cm	r/%	*d*_*t*_/cm	*d*_*e*_/cm	r/%	*d*_*t*_/cm	*d*_*e*_/cm	r/%	*d*_*t*_/cm	*d*_*e*_/cm	r/%	*d*_*t*_/cm
1m	2.24	24.93	(0.89)	1.50	8.32	(1.21)	4.31	15.97	(4.30)	1.79	4.99	(1.41)
2m	2.09	23.25	0.52	1.03	5.73	0.19	2.04	7.56	1.95	1.01	2.80	0.49
3m	2.32	25.79	1.60	1.70	9.43	(1.56)	1.49	5.52	(1.24)	1.03	2.87	(0.67)
4m	3.46	38.44	2.69	2.18	12.11	(1.28)	1.62	6.01	(1.31)	2.52	7.01	1.81
5m	4.55	50.58	3.43	3.19	17.73	0.29	3.96	14.67	1.56	5.50	15.27	4.87

### Performance analysis influenced by the maize planting design

**Fig 6:**
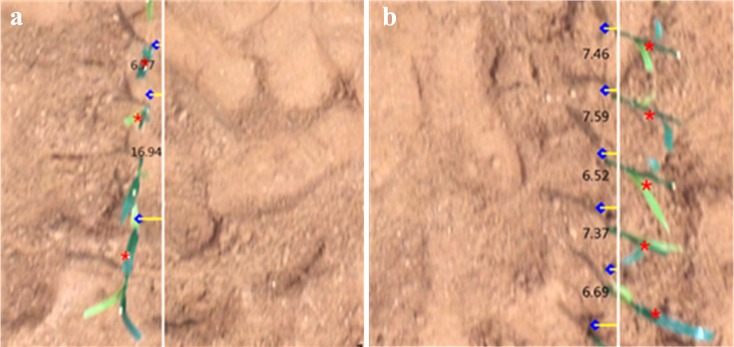
Enlarged view of Fig 1 and related estimated distance of maize intervals. (a) Leaves inteact, and (b) leaves separate.

From these results we conclude that the estimation of the maize interval distance between plants was stable, except for the smallest planting distance (9cm). The *S*_9cm_im_ (i is 1, 2, 3 or 4) for the 9cm spacing were in the lower 10% accuracy of *r*, as shown in Table 2. Fig 6 shows the details of the airborne image and related estimated distance from an enlarged image of a row of artificial plants. Fig 6A shows the performance compared to Fig 6B, where a significant estimated error occurs at 16.94cm, which is beyond the tolerance of application. It was clear that this occurred where leaves of two maize plants overlapped from the vantage point of the camera, resulting in overestimation of the maize interval distance. There are two parameters that could have contributed to the interaction of leaves: 1) a closer distance of the maize interval, or 2) a lowering the camera elevation. While the first possibility is simplistic; the second could form by the shadowing caused by leaves of one plant upon another when the camera’s elevation is low. Fig 6A verifies this. For the *S*_9cm_im_, larger error percentages occurred for all the camera height variations tested because of the close distance of maize interval (9cm).

For other scenarios, most of the relative accuracy metrics, *r*(s), were lower than 10%, which indicates that better performance could be achieved under such conditions. In contrast to the *S*_9cm_im_, the larger maize distance allows enough separation between plants, which is the basis by which individual maize plant objects are extracted.

### Performance analysis influenced by camera height

**Fig 7:**
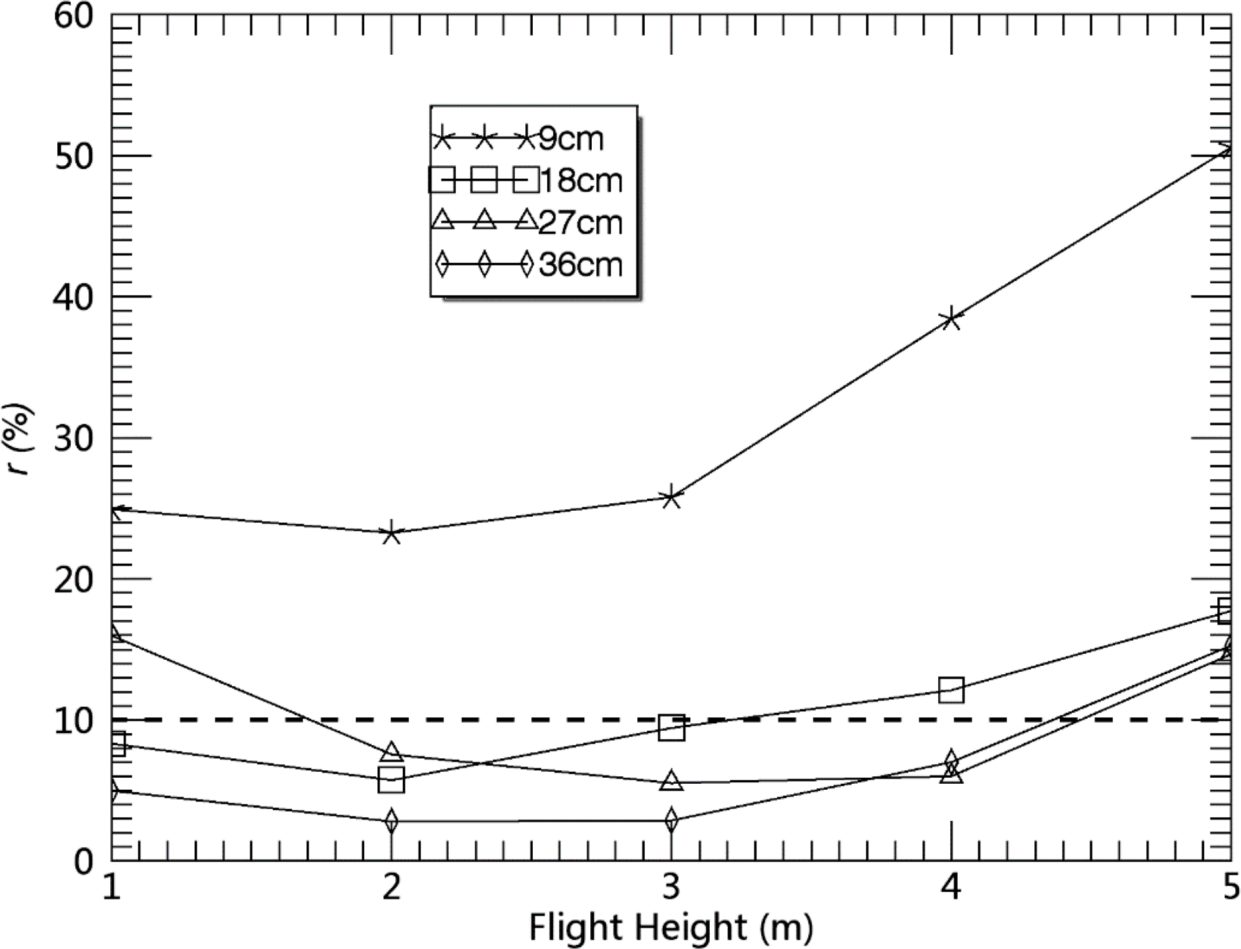
Relative error of crop distance interval estimation.

Fig 7 shows the accuracy variance of maize interval estimation among different camera heights. The error trend of estimation rises with the accompaniment of the flight height and maize intervals. The estimated accuracy of maize interval for *S*_9cm_im_ was lowest, as previously noted.

**Fig 8:**
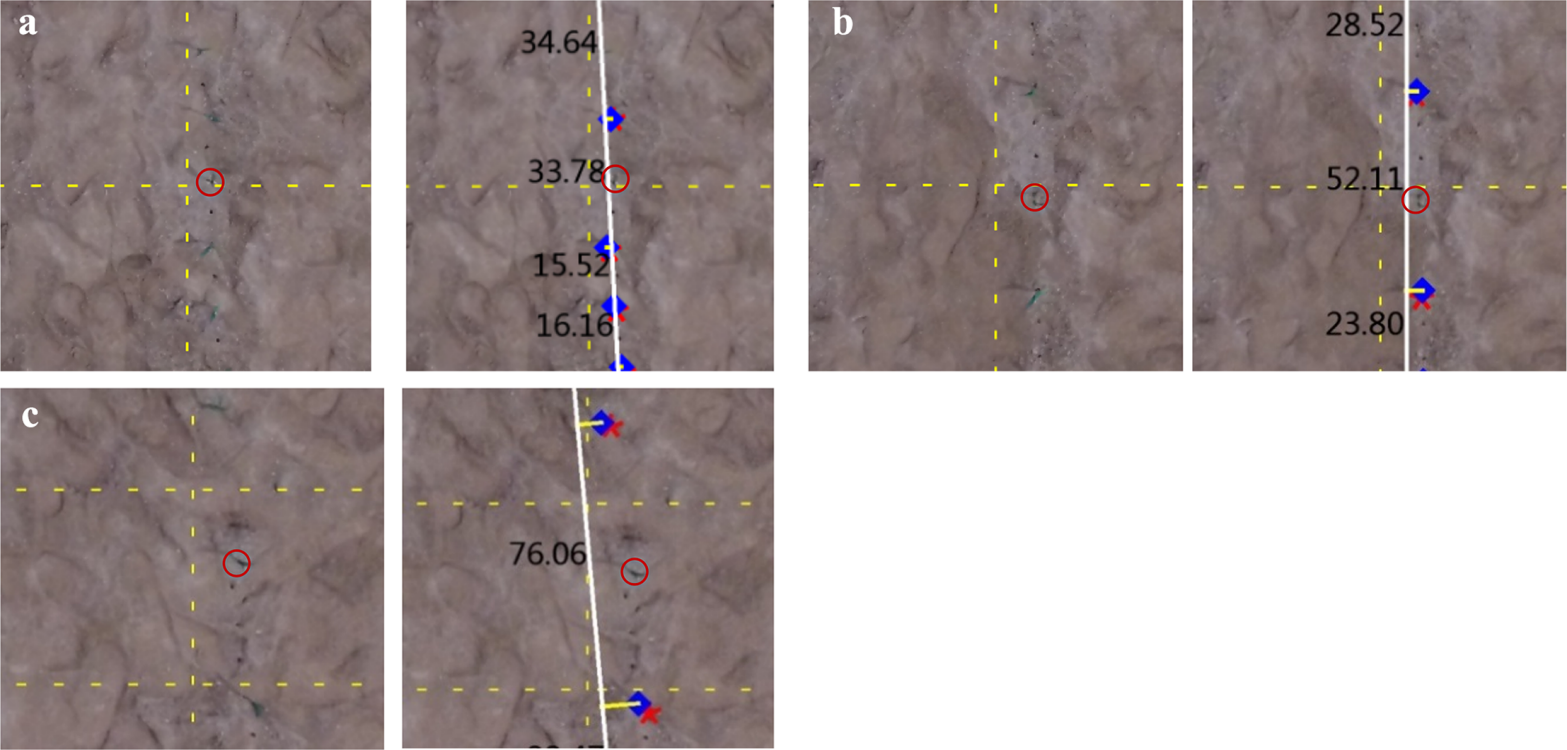
UAV Image at *S*_icm_5m_. **The letter *i* indicates different distances of maize interval.** (a) *S*_18cm_5m,_ (b) *S*_27cm_5m_, and (c) *S*_36cm_5m_.

The S_18cm_im_, *S*_27cm_im_ and *S*_36cm_im_ maize interval distances were chosen to compare the relationship of distance estimation performance to camera height (Fig 8). Blue points represent locations where individual maize plants emerge from the ground. Increased camera elevation creates a narrower angle of view (AOV) and individual plants (maize objects) cover a smaller area in the image. In addition, because individual maize objects cover a smaller area in these images, their identification is obscured by the surrounding land surface signals. Fig 8 shows the significant number of maize objects that were missed (marked as red circles), leading to an overestimation of the maize interval distance. Missed maize objects were small and hard for the camera to capture because the plant reflectance is overwhelmed by the reflectance of the surrounding area. Maize objects tended to be removed from the object collection at these elevations, according to *O*_*maize*_ criteria. In another instance, a number of maize plants appeared small from a certain camera angle, but the object captured in the image only covered a small area. Thus the extent of one scene is larger when the camera is higher. However, the higher elevation causes maize pixel signals captured in the image to fade drastically when compared to images taken at lower camera elevations. The flight height affects the distance estimation of the maize interval. From the viewpoint of the camera, this issue could be solved if the extent of the image was enlarged.

## Experiment 2: Maize planting estimation in the field

The first experiment showed that we could accurately measure maize interval distances from airborne images. under artificial conditions In order to evaluate our method under real-world conditions, a typical maize field (situated at 42.86° W, 85.06° N) was selected for field experimentation. The farmer uses a high-density maize planting strategy with maize row widths of 53 cm and maize interval widths of approximately 20 cm. Maize planting was completed with a planter three weeks prior to the field trials. and residue from the previous season’s cover crop was observable within the field. We flew the UAV at 12:00 PM on June 10, 2016, when the plants were at V4 stage. A broad spectrum herbicide had been applied to the field several days prior to the flights, thus removing a majority of the weeds. We measured the heights of 10 individual maize plants to determine a uniform parameter of 0.15m (the average measured result) for the entire study area.

**Fig 9:**
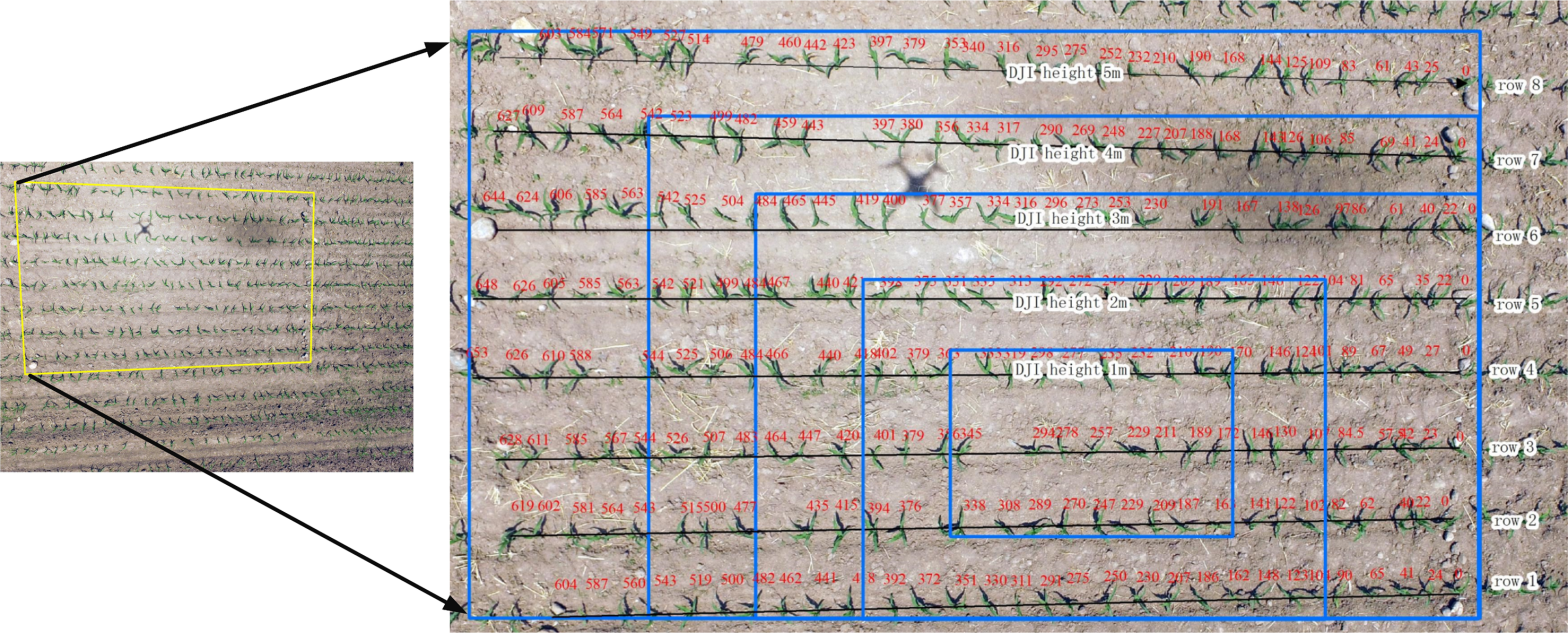
Flight and related plot area covered by UAV imagery.

The plot was designed to cover an area of 6m by 8 rows. A stake was placed at the first and last maize plant of each individually measured test row to serve as a marker. As with the preliminary study on artificial maize plants, UAV flight heights were set at 1m, 2m, 3m, 4m, 5m. Fig 9 shows the plot position and the areas of the images captured in the tested plot based on these camera heights. Blue frames represent the area covered in the plot at each elevation. Red numbers above each maize plant are the maize interval distance (in cm) relative to the starting plant at the right ridge.

The image collection scenario is fundamentally different in a natural environment than indoors with artificial plants. First, the reflectance of actual maize was more complicated compared to its background. We determined that the threshold, 0, was not sufficient to extract the vegetation from the background information. We initially considered maize and weeds as one category due to similarities in their reflectance. Thresholds of 0.01 were attempted for pictures taken at 1m and 0.1m for pictures taken at camera heights of 2m, 3m, 4m, and 5m. This proved to be effective for distinguishing maize from the background reflectance values. Thresholds were varied for the different camera heights, mainly because the light variation caused variation of the land surface brightness.

Another challenge was to distinguish the maize signature from that of weeds. In Fig 9, weed patch clusters and scatters in the field are visible. Weeds vary in size, sometimes grow in clusters and have different growth patterns. The principal of filtering plants by shape doesn’t work when the extracted weeds patches from the threshold-based method are similar in size and shape to maize. Most of the weeds were between the maize plant row ridges. Therefore, we formulated a new condition to filter for weeds based on the assumption that maize exists within a “buffer region” of the maize planting row. If some maize plants were beyond the buffer area, those plants could be considered weeds and were removed from the maize data collection. The remaining maize plants were considered as the basis for interval distance calculation. Weeds growing within a row that resemble maize in height and growth habit could not be excluded from the maize segmentation.

The accuracy assessment is similar to the process used for the artificial maize plants using Eq (17). In order to assess the maize distribution pattern, the accuracy index is calculated row by row as follows:

$$d_{t}=\left(\frac{\sum_{i=1}^{n}ed_{i}}{n}-\frac{\sum_{i=1}^{n'}d_{i}}{n'}\right)$$ (17)

In Eq (17), *d*_t_ is the difference between the estimated distance and the measured distance. Here, *n* and *n'* are the amount of estimated maize and measured maize, respectively. This equation is used to quantiy the miaze distance interval accuracy. Referencing Eq (15), the relative error can be further explained as:

$$r=d_{t}/\frac{\sum_{i=1}^{n'}d_{i}}{n'}$$ (18)

**Fig 10:**
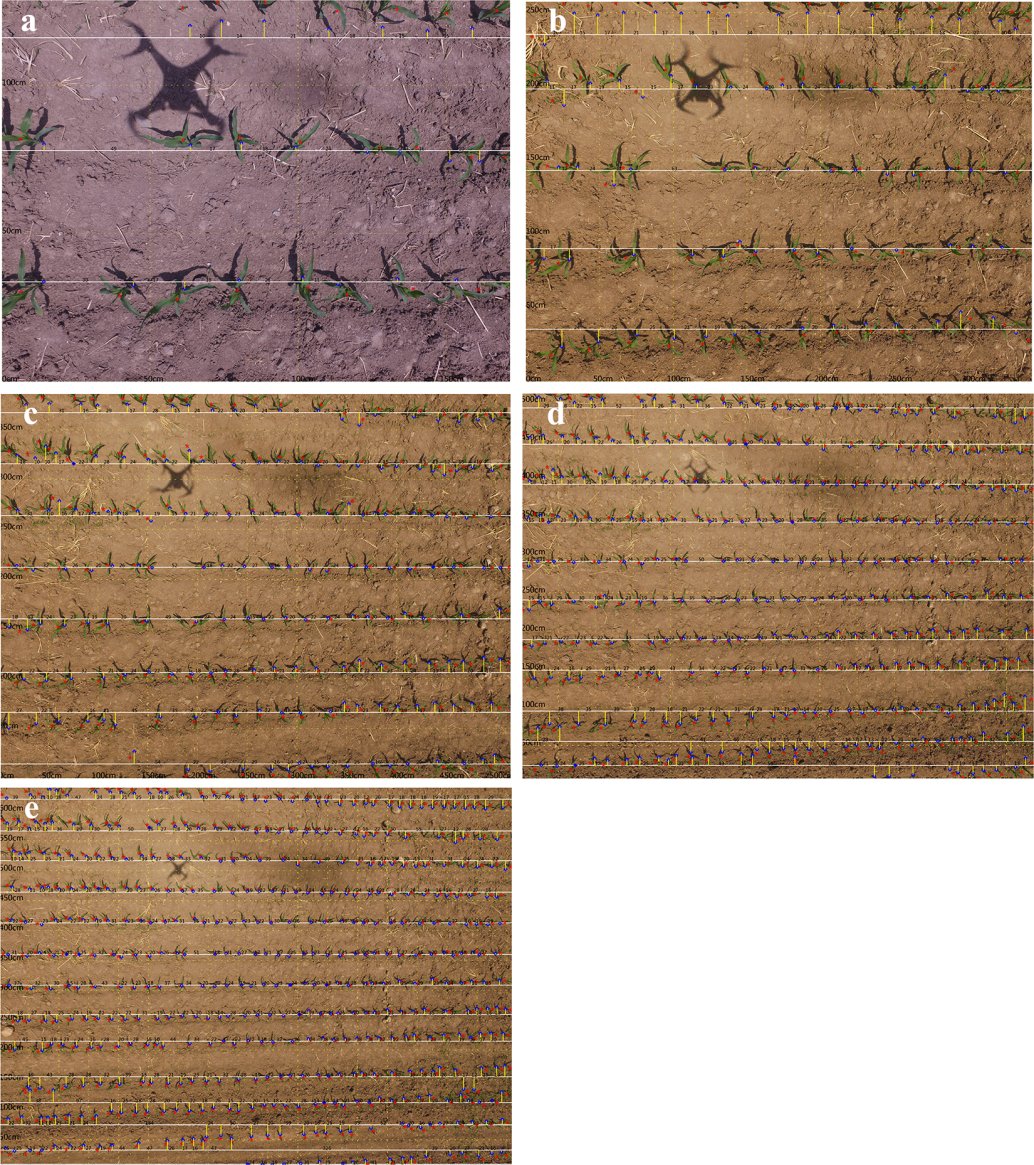
Estimated maize distance with different flight heights. (a) 1m, (b) 2cm, (c) 3m, (d) 4m, and (e) 5m.

From Table 3, except for the flight of 1m, a reliable and convincing performance could achieve a related error of about 3cm, and relative errors of about 10%, which met the requirements of precision agriculture management. Below were results of different flight heights as shown in Fig 10.

**Table 3:** Accuracy assessment of average crop interval distance.

Height		*d*_t_ (cm)							
		row 1	row 2	row 3	row 4	row 5	row 6	row 7	row 8
1m	*d(cm)*	-5.36	-3.70	-1.97					
	*r(%)*	-25.21	-14.95	-8.99					
2m	*d(cm)*	-1.04	-3.47	0.69	1.45	1.43			
	*r(%)*	-4.97	-16.16	3.06	6.44	6.97			
3m	*d(cm)*	2.33	-2.12	-2.15	-2.63	2.90	0.77		
	*r(%)*	11.02	-9.99	-10.14	-10.19	13.33	3.67		
4m	*d(cm)*	2.31	2.63	-0.91	1.17	-0.11	0.35	1.95	
	*r(%)*	10.61	12.15	-4.21	5.59	-0.48	1.54	9.32	
5m	*d(cm)*	0.51	1.81	2.74	1.00	1.56	0.40	2.03	0.65
	*r(%)*	2.43	8.38	12.75	4.79	7.18	1.85	9.20	3.11

The proposed method locates individual maize plants accurately (Table 3 and Fig 10). An interesting finding is that more reliable results can be achieved with higher flight altitudes. This is mainly due to the confusion generated from the overlap of plant leaves that is experienced at lower flight altitude.

Measurement of maize interval distance at 1m flight differs from the other elevations in that the accuracy at this height was relatively low, *r* = -25.21 and *d*_t_ = -5.36. From Fig 10A, the main contribution to this phenomenon may be that the area captured by one photo is relatively small. Some maize plants were truncated and plant residue from the previous season was still in the field, which leads to the deviation in calculating the central points of the maize segmentation when we set a constant maize plant height. This negative factor could be remedied at higher elevations. Another factor to be considered is the overlap of leaves when flying at lower elevations. In Fig 10A, a large distance of the maize interval, 51cm, could be measured in the yellow rectangle. The UAV-based method calculated the interval distance as 49 cm; a 2cm difference, suggesting the proposed method has demonstrated the potential to accurately estimate the maize interval distance.

**Fig 11:**
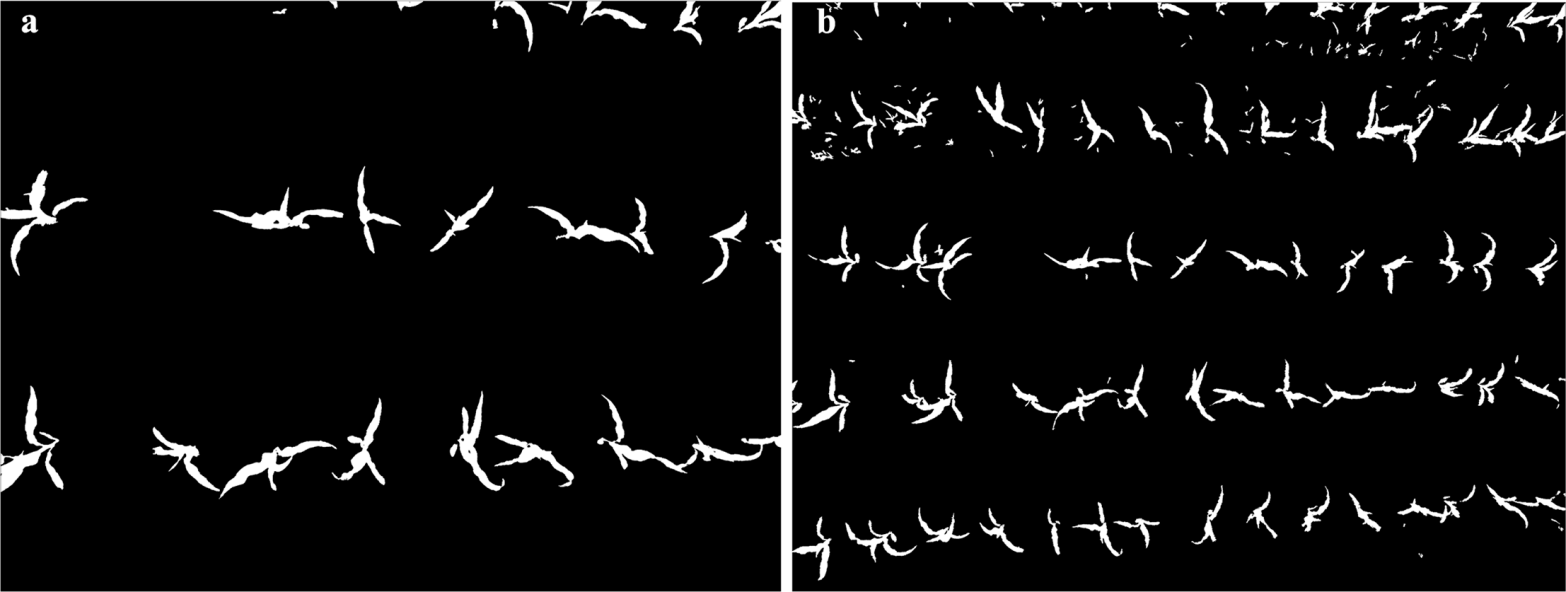
Maize segmentation from the threshold based method at different heights. (a) 1m, and (b) 2m.

According to the proposed method, maize segmentation is the basis for maize interval distance estimation. Fig 11 shows two cases of maize segmentation results at 1m and 2m height, generated from the images in Fig 10A and 10B. Segmentation results demonstrated the maize distribution clearly and accurately, highlighting the effectiveness of the threshold-based method. In Fig 11B, weed patches are considered as maize plants before filtering and represents a major issue to confront. After the filter operation of limited geometry and ridge buffer operation, the weeds were eliminated effectively (Fig 10B). Unfortunately, some weeds will still be counted as maize if the weeds are within the maize row adding to the error of interval distance estimation.

There are limits to defining the crop planting row. In Fig 10E, the maize planting row was defined incorrectly because the maize row direction was not horizontal from west to east, and the predefined setting of zero degrees of maize direction resulted in problematic interpretations.

## Discussion

In this paper we presented a UAV-based image-processing algorithm that was developed to calculate maize plant distances. Knowing the exact number of plants per square meter at emergenceis an essential parameter to help understand and predictyield variability, and to improve variable rate fertilizer and pesticide applications to match plants demand with specific input application [1]. Several studies have clearly demonstrated that plant population is one of the most important factors affecting final grain yield [2–6]. equipmentneverPlant population influences several processes, ranging from soil water balance (soil evaporation, plant transpiration, surface runoff etc.), to nutrient cycling and resource use efficiency (water and nitrogen). A lower number of plants per square meter loses more water from soil evaporation than a crop with higher plant population where more water would be used by the plants, rather being lost from evaporation. Furthermore, lower plant populations allow weeds to invade the available free space available, compared to areas where space is already occupied by crops. All these feedback mechanisms are critical to identify causes of yield variation at field scale. Despite advancements in machinery and planting equipment, intended plant populations seldom reflect true plant populations at emergence. In this paper, we showed that our new method accurately quantifies the distance between crop plants using available UAV technology.

A major problem for the performance of the algorithm is the presence of weeds. This has been resolved by adapting the threshold of two indices: the combination of area and shape parameters to distinguish maize from other vegetation, and the distance tolerance to remove weeds growing beyond a certain distance to the maize row. These combined conditions outperformed the Xavier’s process based on the binary pixel value accumulation. However, weeds growing within crop rows remains a problem that requires further work to resolve.

In our method, plant height is a critical parameter for plant distance calculation. We determined the average height of randomly sampled plants for all plants within images. This was effective for small-area applications since big phenotypic differences were not observed within the area of one UAV image taken from 1 to 5 meters due to the early stage of plant growth. Taking images later in season, where when plant height may vary greatly, would be problematic because the plant leaves would overlap, creating cofounding errors. For this reason, we took our measurements when plants werewere small (V3), whereand phenotypic differences are minimal. There are still several other issues that need to be addressed. This method is complicated to apply, and relies greatly on multiple predefined parameters including maize row distance, maize height, direction of the crop row, and thresholds of segmentation size and ratio of area to perimeter. An automated method that needs less input parameters needs to be developed to improve the applicability and robustness of the proposed method. Also, the flexibility of crop direction should be tested for the crop row detection using other algorithms, such as the Hough Transformation. The influences of tremendous variations in flight heights on plant distance estimation also require further investigation. Additionally, the suitable time-window of UAV imagery captured for the maize field also needs further exploration. The study remains a critical contribution to the literature, as the evaluation of plant distance after emergence is a very important factor to consider when understanding causes of spatial and temporal variability of crop yield in corn. The distance between plants is also a critical input required by crop models, as the simulation principles of crop growth are based on radiation use efficiency which depends on leaf area and intercepted radiation. Erroneous plant population estimates result in incorrect estimation and forecast of grain yield in corn.

## Conclusions

In this paper, we presented a procedure that was developed to calculate maize interval distance using an UAV. It has been verified successfully by the analysis of both indoor and outdoor experiments that were designed to test artificial and actual maize plants. The proposed method includes maize segmentation based on a threshold method using vegetation indices, filtering the maize segmentation from the effects of background noise and weed pressure, adjusting the center points of maize segmentation according to view angle, and finally estimating the maize row by measuring the maize interval distance.

With the exception of the 1m flight height scenario, both experiments demonstrated a relative error of approximately 10%, which is sufficient to support more accurate and timely estimations of maize populations. The threshold-based method for maize segmentation has shown to be effective in the extraction of maize plants from the background (although weeds are still mixed in the maize collection). The method of adjusting the center point of the maize segmentation to the stem location of the maize is also sufficient, and eliminates the distortion of maize from the point of view of the camera.
